# Mitoxantrone-Loaded Nanoparticles for Magnetically Controlled Tumor Therapy–Induction of Tumor Cell Death, Release of Danger Signals and Activation of Immune Cells

**DOI:** 10.3390/pharmaceutics12100923

**Published:** 2020-09-27

**Authors:** Teresa Ratschker, Laura Egenberger, Magdalena Alev, Lisa Zschiesche, Julia Band, Eveline Schreiber, Benjamin Frey, Anja Derer, Christoph Alexiou, Christina Janko

**Affiliations:** 1 Department of Otorhinolaryngology, Head and Neck Surgery, Section of Experimental Oncology and Nanomedicine (SEON), Else Kröner-Fresenius-Stiftung Professorship, Universitätsklinikum Erlangen, 91054 Erlangen, Germany; teresa.ratschker@fau.de (T.R.); Laura_E@web.de (L.E.); magdalena.alev@fau.de (M.A.); lisa.z@fen-net.de (L.Z.); julia.band@uk-erlangen.de (J.B.); eveline.schreiber@uk-erlangen.de (E.S.); christoph.alexiou@uk-erlangen.de (C.A.); 2 Friedrich-Alexander-Universität Erlangen-Nürnberg (FAU), 91054 Erlangen, Germany; 3 Department of Radiation Oncology, Universitätsklinikum Erlangen, 91054 Erlangen, Germany; benjamin.frey@uk-erlangen.de (B.F.); anja.derer@uk-erlangen.de (A.D.)

**Keywords:** nanomedicine, iron oxide nanoparticles, chemotherapy, magnetic drug targeting, cell death

## Abstract

Stimulating the patient’s immune system represents a promising therapeutic strategy to fight cancer. However, low immunogenicity of the tumor cells within an immune suppressive milieu often leads to weak anti-tumor immune responses. Additionally, the immune system may be impaired by accompanying aggressive chemotherapies. We show that mitoxantrone, bound to superparamagnetic iron oxide nanoparticles (SPIONs) as the transport system, can be magnetically accumulated in adherent HT-29 colon carcinoma cells, thereby inducing the same cell death phenotype as its soluble counterpart, a chemotherapeutic agent and prototypic inductor of immunogenic cell death. The nanoparticle-loaded drug induces cell cycle stop, apoptosis and secondary necrosis in a dose- and time-dependent manner comparable to the free drug. Cell death was accompanied by the release of interleukin-8 and damage-associated molecular patterns (DAMPs) such as HSP70 and ATP, which fostered chemotactic migration of monocytes and maturation of dendritic cells. We furthermore ensured absence of endotoxin contaminations and compatibility with erythrocytes and platelets and investigated the influence on plasma coagulation in vitro. Summarizing, with magnetic enrichment, mitoxantrone can be accumulated at the desired place, sparing healthy peripheral cells and tissues, such as immune cells. Conserving immune competence in cancer patients in the future might allow combined therapeutic approaches with immune therapies (e.g., checkpoint inhibitors).

## 1. Introduction

In recent years, immune therapies have constituted substantial improvements in tumor treatment. However, these therapies often fail due to the low immunogenicity of tumor cells and the surrounding immunosuppressive milieu, both resulting in a weak anti-tumor immune response and enabling immune therapies only in a subgroup of patients [1]. Moreover, the tremendous costs will pose a challenge for the health care system in the future [2,3]. Tumor infiltrating lymphocytes have been found to be a good prognostic marker for many cancers such as melanoma, breast cancer, and ovarian cancer [4]. Especially for colorectal cancer, many study reports describe a beneficial effect of lymphocytic infiltration for survival [5]. The cell death pathway and its preceding intracellular sequence play a major role in determining immunogenicity of a tumor cell during cancer treatment [6]. Several conventional tumor treatments with established drugs have been shown to induce immunogenic cell death (ICD), characterized by the time-dependent release of damage-associated molecular patterns (DAMPs) from dying tumor cells, thereby overcoming the immunosuppressive tumor microenvironment [7]. For instance, ICD can be induced by γ-irradiation, photodynamic therapy and chemotherapeutic drugs like doxorubicin and mitoxantrone (MTO) [8,9]. Due to unspecific distribution in the body, these agents commonly induce severe side effects from alopecia and emesis to leukopenia and anemia [10]. In fact, drug distribution studies have shown that only a fraction of the applied dose reaches the tumor region, while the rest leaks into healthy tissues to cause damage [11,12]. Since the immune system is crucial for inhibition of tumor growth, patients with impaired immune system, such as HIV patients, suffer from tumors (e.g., Kaposi’s sarcoma) more frequently than immunocompetent patients [13]. The efficacy of immunotherapies (e.g., checkpoint inhibitors) has been shown to correlate with mutational burden and the appearance of neoantigens [14], as well as immune cell infiltration into the tumor [15]. In conventional chemotherapy, dose calculations are based on laboratory toxicity studies so that patients may receive the maximum tolerated dose [16]. This strategy has led to the limitation and cure of disease in many patients but is also associated with several complications, e.g., immune deprivation, increasing the risk of life-threatening infections [17]. Some established chemotherapeutics applied for tumor treatment are also used as immune suppressive agents (e.g., cyclophosphamide, methotrexate) in severe autoimmune diseases [18]. To reduce nonspecific drug distribution and increase the success of immune therapy, several multifunctional nanoparticles have been developed for the targeted delivery of ICD inducers recently [19]. For example, ICD-inducing chemotherapeutics such as oxaliplatin or doxorubicin may be transported by nanoparticles, increasing immune response by triggering local ICD of cancer cells [20]. Additionally, the combination of these therapeutics with checkpoint inhibitors or molecules that interfere with the immune suppressive indoleamine 2,3-dioxygenase (IDO) pathway has been shown to further improve therapeutic results [21,22,23]. In all cases, nanoparticles accumulated passively in the tumor region via the enhanced permeation and retention (EPR) effect, which, however, does not lead to satisfactory drug accumulation rates in the tumors [24]. We and others previously utilized superparamagnetic iron oxide nanoparticles (SPIONs) as transporter system to deliver therapeutics magnetically into the tumor [25,26]. Using magnetic accumulation of MTO by SPIONs to the tumor area, our group increased the intratumoral drug levels, thereby enabling the reduction of the therapeutic dose to 5–10% of the dose commonly used in systemic application [26]. With increased drug load in the tumor and reduction of the chemotherapeutic burden in other tissues, immune cells of the blood system were spared from the cytotoxic effects [27]. In tumor-bearing rabbits, the tumor continuously shrank after treatment with MTO loaded SPIONs until it was completely dematerialized after several weeks [26], suggesting an immune-mediated rejection rather than complete tumor lysis by the drug. In a previous study, we found that cell death induced by MTO loaded onto SPIONs was accompanied by the release of danger signals in Jurkat cells, with concomitant activation of dendritic cells [28] with comparable efficacy, as may be observed with administration of the free drug. Based on these earlier findings, we further investigated the effects of MTO loaded on SPIONs in HT-29 colon carcinoma cells and its biocompatibility in blood. We confirmed that it is possible to magnetically guide MTO to the desired location and to induce tumor cell death with immunogenic features.

## 2. Materials and Methods 

### 2.1. Cell Lines and Culture Conditions

The adherent colon carcinoma cell line HT-29 (ATCC/LGC GmbH, Wesel, Germany) was cultivated in McCoy’s 5A medium (Gibco, Carlsbad, CA, USA) with either 10% fetal calf serum (FCS) (Biochrom AG, Berlin, Germany) for magnetic accumulation or cell death kinetics, or 10% Panexin NTA (PAN-Biotech GmbH, Aidenbach, Germany) whenever analyses of DAMPs or cytokines were planned, to minimize interactions with the detection. In preceding experiments, we confirmed that HT-29 cells showed similar behavior, no matter if cultivated in medium containing FCS or Panexin. The monocytic cell line THP-1 (TIB-202; American Type Culture Collection [ATCC], Manassas, VA, USA) was cultured in Roswell Park Memorial Institute (RPMI) 1640 medium supplemented with 2 mM glutamine, 10% FCS (Biochrom AG), 100 U/mL penicillin, and 100 μg/mL streptomycin (Gibco^®^). All cells were cultured in a cell culture incubator (INCOmed, Memmert, Schwabach, Germany) at 37 °C, 5% CO_2_ and 95% humidified air. Cells were regularly checked for mycoplasma contamination using PCR kit Venor^®^GeM (Minerva Biolabs GmbH, Berlin, Germany). Before every experiment, count and viability was analyzed using MUSE^®^ Count & Viability assay kit in MUSE cell Analyzer (Merck-Millipore, Billerica, MA, USA). A cell viability above 95% was required to start an experiment.

### 2.2. Synthesis and Characterization of Superparamagnetic Iron Oxide Nanoparticles (SPIONs)

SPIONs were synthetized according to Zaloga et al. [29]. In short, the SPIONs were produced by co-precipitation, then in situ coated with lauric acid (LA) and subsequently covered by a protein corona of human serum albumin (HSA). For every experiment, 10 μg mitoxantrone (MTO; TEVA Pharma, Ulm, Germany) was freshly loaded onto 242 μg Fe of the SPIONs, corresponding to 200 μg MTO per ml nanoparticle solution. This resulted in SPION^MTO^ particles, as MTO binds strongly to the HSA corona, with a corresponding concentration of 410 µM MTO. Non-loaded SPIONs and drug-loaded SPION^MTO^ were characterized previously for physicochemical features (Table 1) [28,29].

The MTO amount in the figures is given as μM, whereas 0.5 μM SPION^MTO^ corresponds to 0.22 μg/mL MTO loaded on 5.32 μg/mL Fe (according to Table 2). As controls, SPIONs were mixed with the equal amount of H_2_O to receive non-loaded SPIONs in the same concentration. H_2_O-treated cells served as controls.

### 2.3. Endotoxin Content

Endotoxin contamination of SPIONs was quantified using EndoZyme Recombinant Factor C Assay (Hyglos, Bernried, Germany), representing an endpoint fluorescent microplate assay intended for in vitro quantitative determination of endotoxins in pharmaceuticals and biological substances. Importantly, false positive reactions by β-glucan reactions (as occasionally occurring in the Limulus Amebocyte Lysate test) are thereby eliminated. SPIONs of three different batches were taken up in endotoxin-free water in concentrations of 25 µg/mL and 50 µg/mL and tested in duplicates. Samples spiked with 5 EU/mL endotoxin served as inhibition/enhancement controls (IEC). 100 µL of mixture of assay reagent liquid (containing 80 µL assay buffer, 10 µL substrate and 10 µL enzyme) was added to every sample and standard. The reaction was monitored for 90 min at 37 °C in the Microplate Reader Filter Max F5 (Molecular Devices, Biberach an der Riss, Germany). The endotoxin level in EU/mL per sample was calculated from the standard curve (non-linear 4-parameter logistic regression). The regression coefficient R^2^ of the standard curve was >0.98 and the spiking recovery was between 50 and 200%.

### 2.4. Plasma Coagulation

To analyze whether SPIONs influence plasma coagulation, platelet-poor plasma (PPP) was obtained by centrifugation (15 min, 2500× *g*, room temperature) of freshly drawn sodium citrate-anticoagulated blood from healthy donors with normal coagulation times. Ex vivo use of human blood for nanoparticle testing was approved by the ethics committee of the FAU Erlangen-Nürnberg (ethical approval Nr. 257_14B, approved 18 September 2014 and prolonged 6 February 2018). 450 µL plasma was incubated for 30 min at 37 °C with 50 µL of SPION dilutions, resulting in final iron concentrations of 20, 100, or 500 µg/mL. Addition of 50 µL H_2_O served as negative control. After that, plasma was pipetted into pre-warmed cuvettes, a metal ball was added, and coagulation was started by addition of the respective coagulation reagent to each cuvette. The samples were analyzed regarding activated partial thromboplastin time (aPTT), prothrombin time (PT) and thrombin time (TT), using DiaSys coagulation kits (aPPT, CoaguQuickR and TT DiaSys, Holzheim, Germany) in a blood coagulometer (Coagulometer MC4 plus by Merlin Medical, Lemgo, Germany) according to the manufacturer’s instructions. Standard plasma served as the control for normal and abnormal coagulation times.

### 2.5. Platelet Aggregation

Sodium-heparin anticoagulated blood was collected from healthy volunteers and centrifuged at 200× *g* for 30 min at room temperature to receive platelet-rich plasma (PRP) and at 2500× *g* for 15 min to receive platelet-poor plasma as background control (blank). To ensure normal platelet function, all aggregation assays were performed within 4 h of blood collection. To analyze whether SPIONs induce platelet aggregation, 90 µL PRP was incubated with 10 µL SPION dilutions, resulting in final iron concentrations of 20, 100 and 500 µg/mL. Phosphate-buffered saline (PBS, Sigma Aldrich, St. Louis, MO, USA) served as negative control (NC), H_2_O as vehicle control (VC) and 100 µg/mL collagen as positive control (PC) (Sigma Aldrich, St. Louis, MO, USA). Samples were incubated with continuous shaking for 15 min at 37 °C. Then, 50 µL of each sample were diluted in PBS and analyzed by flow cytometry. Data analysis was performed with KaluzaTM software version 1.2.

### 2.6. Hemolysis

Lithium heparin anti-coagulated blood was taken from healthy donors. Hemoglobin-free plasma was prepared as a control by centrifugation of the blood at 800× *g* for 15 min at room temperature (RT). The hemoglobin content of the whole blood samples was determined and adjusted to 5 mg/mL in PBS. SPIONs were incubated with diluted blood in final iron concentrations of 20, 100 µg/mL for 3 h at 37 °C and carefully mixed every 30 min. 1% Triton X-100 (Carl Roth, Karlsruhe, Germany), PBS and H_2_O served as positive, negative and vehicle controls, respectively. To detect interference of SPIONs with the assay, the positive control (PC) was spiked with SPIONs. SPIONs diluted in H_2_O in the respective concentrations served as background controls. After incubation, the tubes were centrifuged for 15 min at 800× *g* at RT to achieve sedimentation of erythrocytes. The supernatant was transferred into new tubes and centrifuged for 1 h at 18.000× *g* at RT to sediment the SPIONs. To determine the content of free hemoglobin, 100 µL supernatant was transferred into the wells of a 96-well plate and incubated with 100 µL Drabkin’s solution (Sigma Aldrich, St. Louis, MO, USA) for 3–5 min at 56 °C on a heating plate until the content of the wells became clear. Drabkin’s reagent converts unstable released hemoglobin and its derivatives to methemoglobin and then to stable cyanmethemoglobin, which was measured at 590 nm on Microplate Reader Filter Max F5. The absorption values measured from the released hemoglobin of the positive control were set to 100%.

### 2.7. Magnetic Accumulation of SPION^MTO^

1 × 10^5^ HT-29 cells were seeded into 12-well plates and cultured overnight. The next day, a 96-well plate containing magnets was positioned directly under the 12-well plates, so that each cell-containing well possessed one central magnet. The cells were incubated with SPIONs, free MTO, or SPION^MTO^ for 5 h in FCS-containing HT-29 medium. Then, the medium was removed, cells were washed with PBS, fixed with 3% paraformaldehyde (PFA; Carl Roth GmbH & Co. KG, Karlsruhe, Germany) in PBS and stained with 10 µg/mL Hoechst 33342 (Thermo Fisher Scientific, Waltham, MA, USA). Fluorescence microscopy pictures were prepared using Zeiss Axio Observer.Z1 fluorescence microscope (Carl Zeiss AG, Oberkochen, Germany) in tile modus and single tiles were stitched to a complete overview picture. Analysis of MTO distribution was performed with ZEN 2012 software (Blue Edition) (Carl Zeiss AG). 

### 2.8. Determination of Cell Proliferation

HT-29 cells were seeded into 24-well plates with 1 mL FCS-containing HT-29 medium in a concentration of 1 × 10^4^ cells/well. The plates were incubated for adherence for either 72 h or 96 h. After that, the medium was replaced with 1 mL Panexin- or FCS-containing medium and the cells were treated with SPIONs, free MTO or SPION^MTO^. To analyze cell proliferation, an IncuCyte life cell imaging system (Essen BioScience Inc., Ann Arbor, MI, USA) was used. All samples were run in triplicates. From the microscopic pictures the amount of area covered by cells (confluence) was calculated. The plates were observed until 100% confluence of the untreated control group was achieved. Confluence was evaluated with Microsoft Excel. In FCS- and Panexin-containing media, cell proliferation was comparable (data not shown).

### 2.9. Determination of Cell Cycle and Cell Death 

24 h, 48 h or 72 h after treatment, supernatants of the cells were collected and frozen at −80 °C for further use. Then, cells were harvested using 0.05% Trypsin (PAN-Biotech GmbH, Aidenbach, Germany). 25 µL aliquots of the cells were either fixated in ethanol (70%) and frozen at −20 °C for cell cycle analysis or stained for cell viability measurement in flow cytometry. For that purpose, 10 µL of cells were stained with 10 µg Hoechst 33342 (Thermo Fisher Scientific, Waltham, MA, USA), 1 µL AnnexinA5-fluorescein isothiocyanate (FITC) or 1 µL AnnexinA5-allophycocyanin (APC, both from ImmunoTools GmbH, Friesoythe, Germany) and 66.6 ng propidium iodide (PI, Sigma-Aldrich Chemie GmbH, Munich, Germany) per 1 mL Ringer’s solution (Fresenius Kabi, Bad Homburg, Germany) for 30 min at 4 °C.

For cell cycle characterization, the ethanol-fixated cells were washed with PBS and incubated for 5 min with 500 µL DNA extraction buffer (19.2 mL 0.2 M Na_2_HPO_4_, 0.8 mL 0.1% Triton X-100 (Sigma-Aldrich Chemie GmbH, Munich, Germany), pH 7.8) following Riccardi et al. [30]. After that, cells were centrifuged, the supernatants were removed and DNA was stained with 300 µL of DNA staining buffer (20 µg/mL PI, 200 µg/mL RNAse A (Sigma-Aldrich Chemie GmbH, Munich, Germany) in PBS for 30 min at room temperature. PI fluorescence was analyzed by flow cytometry.

### 2.10. Flow Cytometry

Flow cytometry was performed on a Gallios flow cytometer (Beckman Coulter, Fullerton, CA, USA). FITC and PI were excited at 488 nm and fluorescence was detected on fluorescence (FL) 1 sensor, 525/38 nm, bandpass (BP) and FL 3 sensor, 620/30 nm BP, respectively. APC and MTO were excited at 638 nm and fluorescence was detected at FL6, 675/20 nm BP or FL7 sensor, 725/20 nm BP, respectively. Electronic compensation was used to eliminate any fluorescence bleed-through. The data were analyzed in KaluzaTM software version 1.2 (Beckman Coulter, Fullerton, CA, USA) and processed in Microsoft Excel. 

### 2.11. ATP Release

To analyze the amount of ATP in cell supernatants, the ATP Determination kit (Molecular Probes, Eugene, OR, USA) was used according to the manufacturer’s instructions. In brief, supernatants frozen at −80 °C were thawed gradually and mixed. 10 µL were placed in each well of a white 96-well plate (PS, 96 Well, Greiner Bio-One GmbH, Frickenhausen, Germany). Then, 90 µL reaction mix (containing 8.9 mL H2O, 0.5 mL 20 × reaction buffer, 0.1 mL 0.1 M dithiotreitol, 0.5 mL 10 mM d-luciferin and 2.5 µL firefly luciferase 5 mg/mL) was added. The ATP-mediated chemiluminescence was measured after incubating for 40 min at 28 °C employing Microplate Reader Filter Max F5. The ATP content of the samples was quantified using a standard curve ranging from 0.1 pM to 1 µM ATP. The data were analyzed with Microsoft Excel.

### 2.12. Heat Shock Protein 70 (HSP70) Release

Heat Shock Protein 70 (HSP70) in the cell supernatant was determined using the Total HSP70 DuoSet^®^ IC ELISA (R & D systems^®^ Inc., Minneapolis, MN, USA) according to the manufacturer’s instructions. In detail, wells of a flat-bottom high-binding 96-well microplate (Greiner Bio-One GmbH, Frickenhausen, Germany) were coated with 100 µL capture antibody (2.0 µg/mL in PBS) overnight at room temperature. After blocking the plate with 300 µL blocking buffer composed of 1% BSA (Merck KgaA, Darmstadt, Germany) and 0.05% NaN_3_ (Carl Roth GmbH & Co. KG, Karlsruhe, Germany) in PBS for 90 min at room temperature, 100 µL of the samples or standards was pipetted into the wells and incubated for two hours. Subsequently to placing 100 µl of detection antibody (3.6 µg/mL in 1% BSA in PBS) into the wells and incubating for two hours, 100 µL of Streptavidin-HRP (50 µL in 10 mL in 1% BSA in PBS) was pipetted into the wells and incubated at room temperature for 20 min. Between each step the plate was washed with 200 µL 0.05% Tween20 (Carl Roth GmbH & Co. KG, Karlsruhe, Germany) in PBS. Finally, the plates were developed by adding 100 µL freshly prepared substrate solution (Carl Roth GmbH & Co. KG, Karlsruhe, Germany) consisting of 1 mL of 0.01 g 3,3‘,5,5‘-tetramethylbenzidine (Sarstedt AG & Co. KG, Nümbrecht, Germany) dissolved in 10 mL dimethyl sulfoxide, 9 mL of 0.1 M Na_2_HPO_4_, 0.05 M citric acid, pH 4.5-5.5 and 2 µL H_2_O_2_). The plate was incubated for 20 min and reaction was stopped with 100 µL of 2N H_2_SO_4_ (Carl Roth GmbH & Co. KG, Karlsruhe, Germany). 170 µL of the samples were then transferred into a clear 96-well plate (Sarstedt AG & Co. KG, Nümbrecht, Germany) and measured in Microplate Reader Filter Max F5 at 450 nm. The HSP70 content in the samples was quantified using a standard curve ranging from 312.5 µg/mL to 10 mg/mL HSP70. The data were analyzed with Microsoft Excel.

### 2.13. Chemotaxis 

The chemotactic capacity of supernatants from HT-29 cells treated with SPION^MTO^ was assessed in a 96-well Chemo-Tx plate (NeuroProbe, Gaithersburg, MD, USA). In brief, the microplate wells were filled with 30 µL of supernatant. MCP-1 (50 ng/mL, Peprotech, Hamburg, Germany) was used as a positive control. After placing the filter frame, the filter top sites with 5 µm pores were filled with 25 µL of THP-1 monocytes at a concentration of 1 × 10^6^ cells/mL. Following incubation for 1 h at 37 °C, migrated non-adherent cells from the lower wells were fixed and counted using flow cytometry. All samples were run in quadruple and averaged. The mean number of migrated cells in the negative control (MCP-1 unstimulated) samples was set as 1.

### 2.14. Generation of Human Immature Dendritic Cells (iDCs)

After giving informed consent, healthy volunteers provided leukocytes in human leukoreduction system chambers (approved by the ethics committee of the Friedrich-Alexander-Universität Erlangen-Nürnberg # 180_13 B, approved 11 September 2013 and 48_19B, approved 19 March 2019). As described by Schaft et al. [31], monocyte-derived immature dendritic cells (iDCs) were differentiated from human blood cells. To summarize, peripheral blood mononuclear cells (PBMCs) were isolated via density gradient centrifugation using Lymphoflot (Bio-Rad, München, Germany) by carefully layering 10 mL blood cells onto 15 mL Lymphoflot in four 50 mL falcon tubes (Sarstedt, Nümbrecht, Germany) and centrifuging the tubes at 850× *g* for 25 min at room temperature without brakes. The PBMCs were washed three times in PBS containing 2% EDTA (Carl Roth, Carlsruhe, Germany) and resuspended in DC medium composed of RPMI medium (Lonza, Basel, Switzerland) supplemented with 1% heat inactivated 0.22 µm filtered human AB-plasma (Sigma-Aldrich, USA), 1% l-Glutamine, and 0.4 µg/mL Gentamycin (both Gibco, ThermoFisher Scientific, Waltham, MA, USA). After seeding the cells in cell culture dishes (4 × 10^7^ cells/dish; BD Biosciences, San Jose, CA, USA) and incubating them for 1.5 h at 37 °C for adherence, non-adherent cells were removed and 10 mL DC medium was added to the remaining adherent monocytes. Further incubation for 6 days with recurring fresh DC medium substitution (2 mL on day 1, 4 mL on days 3 and 5), containing 275 U/mL IL-4 and 800 U/mL GM-CSF (both from Miltenyi Biotec, Bergisch Gladbach, Germany) generated the iDCs.

### 2.15. Maturation of Human Dendritic Cells with Supernatants of HT-29 Tumor Cells

4 × 10^5^ HT-29 cells cultivated with 6 mL Panexin-medium in T 25 cell culture flasks (Sigma-Aldrich, USA) were treated with 1 µM or 5 µM SPION, MTO and SPION^MTO^ as described above. After 72 h, the supernatants were separated from the cells and frozen at −80°C for further experiments. On day 6, 100 µL DC medium containing 1 × 10^6^ iDCs, which were harvested from the dishes, centrifuged at 200× *g* for 12 min and resuspended in 10 mL DC medium, were added to 2 mL HT29 cell supernatant per well of a 6-well plate. For every sample at least one duplicate was prepared. To generate mature DCs (mDCs), a cytokine cocktail containing IL-1β (200 U/mL), IL-6 (1000 U/mL), TNF-α (10 ng/mL, all from ImmunoTools, Friesoythe, Germany), and PGE2 (1 µg/mL, Pfizer, New York, USA) in DC medium served as a positive control. Untreated DCs served as negative immature DC controls (iDCs). After 24 h of co-incubation, supernatants of DCs were frozen for further cytokine analysis at −80 °C. DCs were harvested, washed and adjusted to a density of 5 × 10^4^ cells per 50 µL in ice-cold PBS containing 2% FCS. Staining for DC activation markers was performed with fluorochrome-conjugated antibodies and their appropriate isotype controls for 30 min at 4 °C, using CD197-PE, CD25-FITC, CD83-PE/cy7, CD40-PerCP/Cy5.5, CD209-APC, CD80-APC, CD86-PerCP/Cy5.5, CD70-PE (all from BioLegend, San Diego, CA, USA) and HLA-DR-eFluor450 (ThermoFisher Scientific, Waltham, MA, USA). Stained DCs were analyzed in flow cytometry (Gallios, Beckman Coulter, Fullerton, CA, USA). We identified DCs using the forward and side scatter properties and excluded CD3, CD14, CD16, CD19, CD20, CD56 staining typical for T cells, monocytes, granulocytes, B cells and NK cells, respectively. The gated cell population was positive for CD11c and CD1c, both known markers for DCs. Data were analyzed using KaluzaTM software version 1.2. 

### 2.16. Cytokine Analysis 

Cytokines released from DCs were analyzed using the LEGENDplex human IL-8 detection kit or the LEGENDplex human inflammatory panel (both from BioLegend, San Diego, CA, USA) according to the manufacturer’s instructions. In short, 10 µL of assay buffer, 10 µL of supernatant, 10 µL mixed beats and 10 µL detection antibody were combined in small tubes (Micronic, Lelystad, Netherlands) and incubated at room temperature, in the dark, on a plate shaker for two hours. Then, 10 µL Streptavidin-phycoerythrin was added and incubated for further 30 min. Next, the tubes were centrifuged at 1000× *g* for 5 min and the supernatant was removed. The pellet was washed with 300 µL Ringer’s solution and finally resuspended in 150 µL Ringer’s solution. The samples were measured in flow cytometry with excitation at 488 nm and 638 nm and detection in FL2 (575/30 BP) and FL6 (675/20 BP). LEGENDplexTM data analysis software was used to analyze the data.

### 2.17. Data Analysis and Statistics

Data were processed in Microsoft Excel (Microsoft, Redmond, WA, USA). Graphs and statistics were prepared in GraphPad Prism software, version 8 (San Diego, CA, USA). Group differences were calculated after performing a normality test (Shapiro-Wilk), using Student’s *t*-test in the samples passing the normality test and non-parametric Mann–Whitney U test in those failing.

## 3. Results

### 3.1. Biocompatibility

**Figure 1:**
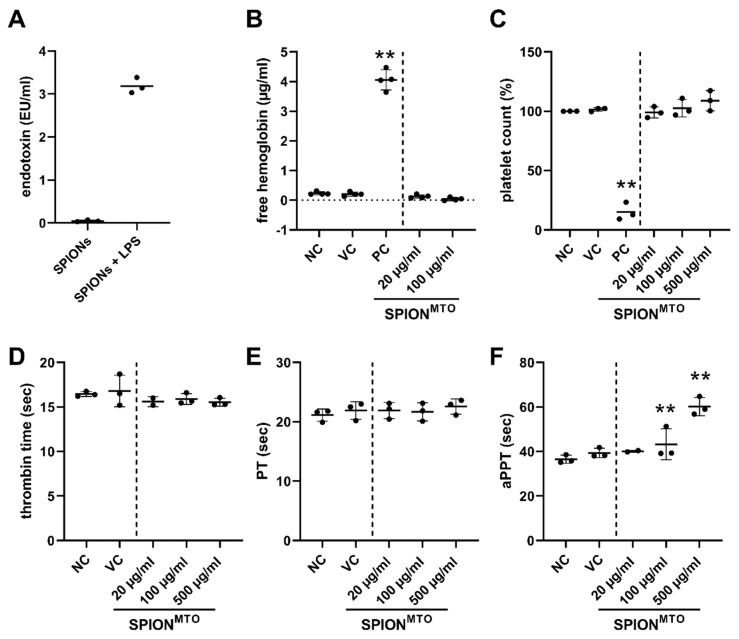
Blood compatibility of SPIONs. (**A**) Endotoxin content. Three batches of SPIONs were analyzed for endotoxin contaminations. Shown are the mean values with standard deviations of the three batches tested in duplicates of 25 µg/mL iron content. For exclusion of assay interference, SPIONs were spiked with 5.0 EU/mL lipopolysaccharide (LPS) and spiking recovery was determined. (**B**) Hemolysis. Anticoagulated blood was incubated with SPIONs for 30 min. Then, the samples were centrifuged to remove erythrocytes and nanoparticles. Free hemoglobin in the supernatant was detected at 590 nm, reflecting hemolysis. Samples treated with Triton X-100 served as positive control (PC). (**C**) Platelet aggregation. Platelet-rich plasma was incubated with SPIONs for 15 min under continuous shaking. The number of platelets was measured by flow cytometry. Collagen-treated samples served as positive control (PC). (**D**–**F**) Plasma coagulation time. Human anticoagulated platelet-poor plasma was incubated with SPIONs for 30 min and then analyzed for coagulation. (**D**) thrombin time (TT), (**E**) prothrombin time (PT), (**F**) activated partial thromboplastin time (aPPT). Indicated concentrations refer to the iron content in the samples. (B-F) Experiments were performed in triplicates of blood from 3 healthy donors, every dot represents the mean values of one donor. The line indicates the mean values of all donors with standard deviations (** *p* ≤ 0.01; treated samples versus negative control, NC). In all samples, PBS served as negative control (NC), water as vehicle control (VC), the positive controls (PC) were selected individually for each readout.

SPION^MTO^ are intended for use in magnetic drug targeting, implying that after intraarterial application, they will come into close contact with components of the blood. Therefore, it is mandatory that the particles are free of endotoxins to prevent immune stimulation. To exclude assay interference by the nanoparticles, the samples were spiked with endotoxin and spiking recovery was determined. Dilution of the SPIONs to 25 µg/mL Fe content resulted in an acceptable endotoxin spiking recovery of 64%. For 25 µg/mL <0.05 EU/mL endotoxin was detected, showing that the nanoparticles are eligible for further investigations (Figure 1A). Since the used MTO was of pharmaceutic quality and loading was strictly performed under aseptic sterile conditions immediately preceding every experiment, sterility and absence of endotoxins was also assumed for SPION^MTO^. To ensure hemocompatibility, we analyzed the effect of SPIONs on erythrocytes, aggregation of platelets and plasma coagulation. We investigated SPION^MTO^ in iron amounts of 20, 100, 500 µg/mL, whereas 20 µg/mL is approximately the iron amount corresponding to 2 µM MTO in the SPION^MTO^ samples (Table 2). Thus, the doses tested were rather high to ensure their biocompatibility.

Damage of the erythrocyte membrane leads to release of hemoglobin, which is toxic when present in the extracellular space and can cause life-threatening situations [32]. To analyze hemocompatibility, we performed quantitative colorimetric determination of free hemoglobin of erythrocytes co-incubated with SPIONs as previously described [33]. Compared to the positive control (Triton X-100), the incubation of blood from three donors with SPIONs did not induce release of hemoglobin, even in the highest tested SPION concentration (Figure 1B).

Next, we investigated the effect of SPION^MTO^ on platelets, which play a key role in hemostasis. Engineered nanomaterials may change the platelet number or function leading to abnormal bleeding or predisposition for thrombosis [34]. Platelet-rich plasma incubated with nanoparticles showed no decrease in free platelets, which would indicate platelet aggregation (Figure 1C).

Proteins of the coagulation system, once activated, act in a cascade of enzymatic events leading to blood clotting which prevents blood loss after injury. However, activation of this cascade by nanomaterials can also lead to unwanted occlusion of vessels. Thus, we investigated whether SPIONs impair plasma coagulation in vitro. Plasma was incubated with nanoparticles and clot formation was analyzed. We found that SPIONs did not alter thrombin time (Figure 1D) and prothrombin time (PT) (Figure 1E) in iron concentrations up to 500 µg/mL. However, the activated partial thromboplastin time (aPPT) was significantly prolonged by the nanoparticles in a dose-dependent manner (Figure 1F).

### 3.2. Magnetic Accumulation of SPION^MTO^

**Figure 2:**
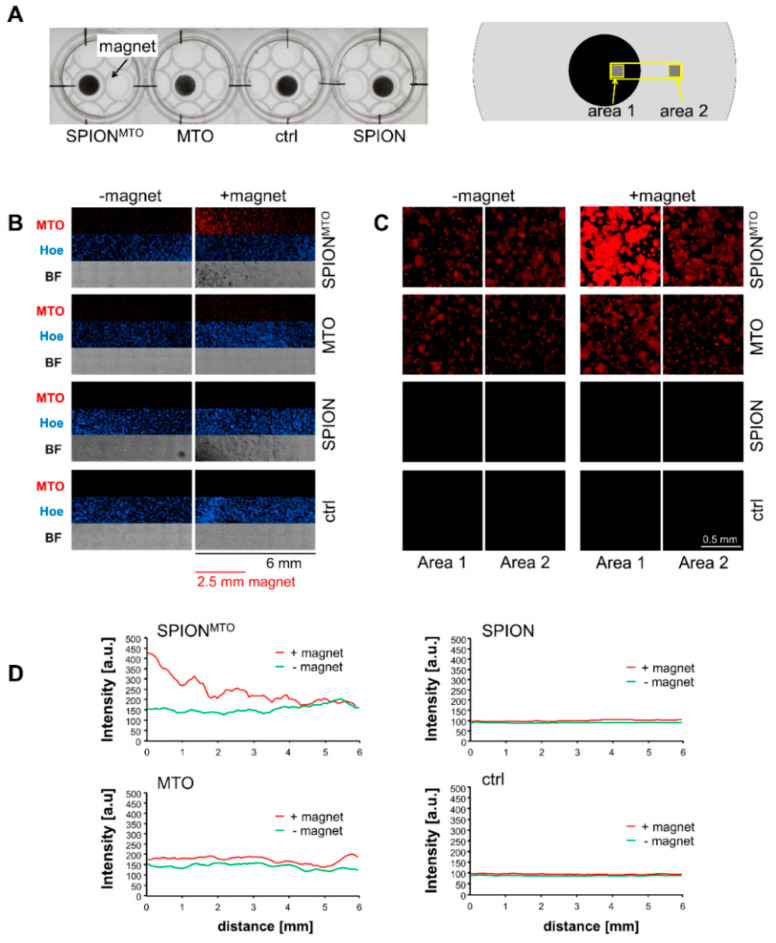
Magnetic accumulation. (**A**) Experimental setup: HT-29 cells seeded into 12-well plates were treated with free MTO, SPION^MTO^ and unloaded SPIONs (MTO concentration 0.25 µM with corresponding Fe concentration, see Table 2). Magnets (diameter 0.5 cm) were placed under the cell-containing plate and incubated for 5 h. (**B**) Overview pictures. Cells were fixated and stained with Hoechst 33342. 8 × 3 single pictures were taken in fluorescence microscopy and stitched together, resulting in a 6 mm × 1.33 mm large area (see yellow box in (**A**), right scheme). (**C**) Close-up on areas 1 (cells above the magnet) and 2 (cells in distance to the magnet). (**D**) Distribution of MTO fluorescence along the analyzed area was determined with Zen software. BF: Bright field images. The experiment was performed in triplicates.

To monitor MTO accumulation in vitro, HT-29 cells were treated with MTO, SPION or SPION^MTO^ in wells (2.1 cm diameter) with magnets (0.5 cm diameter) positioned under the cell-containing wells (Figure 2A). Since MTO has an intrinsic fluorescence, drug accumulation can easily be monitored via fluorescence microscopy. Using the stitching function, single images were merged so that a total area of approximately 6 mm × 1.5 mm was analyzed (Figure 2B). Comparing the distribution of MTO in its free and nanoparticle-bound form in the presence and the absence of a magnetic field revealed no difference in the absence of a magnetic field, whereas nanoparticle-bound MTO clearly accumulated over the magnet (Figure 2B). Staining with Hoechst 33342 showed an even distribution of the cells. Transmission microscopy pictures indicated enrichment of SPION^MTO^ and SPION in the presence of a magnet, whereas in the absence of magnets, no particle enrichment was detectable. Further magnifications of area 1 and area 2 (Figure 2A right) illustrated the cellular localization and cellular accumulation of the drug in case of nanoparticle loading in the presence of a strong magnetic field (area 1) compared to sites located farther from the magnet with a weak magnetic field (area 2) (Figure 2C). The analysis of MTO fluorescence in the depicted region also indicated a gradient-like enrichment of MTO-loaded nanoparticles in the presence of a corresponding magnetic field gradient, whereas the distribution of free MTO is magnetically independent (Figure 2D).

### 3.3. Drug Uptake and Cell Death Induction

**Figure 3:**
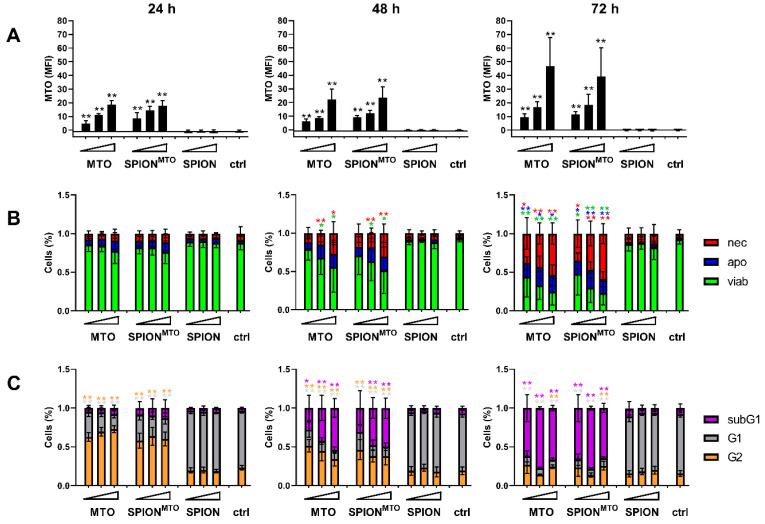
MTO uptake, cell cycle and cell death induction. HT-29 cells were treated with SPIONs, MTO, or SPION^MTO^ for 24 h, 48 h, and 72 h. MTO concentrations of 0.5, 1.0 or 2.0 µM (see triangle) and equivalent SPION concentrations were administered according to Table 2. H_2_O-treated cells served as controls. (**A**,**B**) Cells were stained with Annexin A5-FITC or -APC (Ax) and propidium iodide (PI) and analyzed via flow cytometry. (**A**) The mean fluorescence intensity (MFI) of viable cells (Ax^−^ PI^−^) was used to quantify intracellular MTO. (**B**) Ax^−^ PI^−^ cells are considered viable (viab), Ax^+^ PI^−^ are considered apoptotic (apo) and Ax^+^ PI^+^ are considered necrotic (nec). (**C**) Cell cycle was analyzed by PI-Triton staining in flow cytometry. G1 reflect cells with diploid DNA content, G2 reflect cells with double diploid DNA content, and subG1 are cells with degraded DNA. Shown are mean values of three independent triplicates with standard deviations. Significances were calculated using Student´s *t*-test for treatment groups versus control and MTO versus SPION^MTO^ (* *p* < 0.05; ** *p* < 0.01).

To analyze MTO and SPION uptake by HT-29 cells, we analyzed MTO fluorescence and side scatter increase in flow cytometry (Figure 3; Figure S1). Side scatter increase has previously been shown to correspond with nanoparticle uptake and/or attachment into the cells or to the plasma membranes, respectively [35]. Intracellular MTO fluorescence increased over time and as a function of administered drug concentration, showing no significant difference between free MTO and SPION^MTO^. Both the control and the groups treated with unloaded SPIONs displayed no MTO signal (Figure 3A, Figure S1a,c). Instead, cells treated with SPION revealed increased side scatter values, indicating cellular nanoparticle uptake (Figure S1a,b). Furthermore, the cellular morphology also changed under treatment with free or nanoparticle-bound MTO, which might be due to early blebbing processes during cell death. Side scatter values of cells treated with SPION^MTO^ were remarkably higher, probably because they were influenced by both, nanoparticle uptake and cell death processes (Figure S1a,b). In light microscopy, cells treated with MTO or SPION^MTO^ appeared shrunken and detached (Figure S1d). Cells treated with SPION or SPION^MTO^ contained clearly visible brown particles (Figure S1d).

Effects on cell viability were assessed using Annexin A5-FITC (Ax) and propidium iodide (PI) staining. Ax binds phosphatidylserine, which switches from the inner to the outer leaflet of the plasma membrane when cells undergo apoptosis [36]. The plasma membrane impermeable dye PI binds DNA, indicating ruptured plasma membranes and thus implies necrosis. 24 h after treatment with SPION, MTO or SPION^MTO^ only slight dose-dependent effects were detected with AxPI staining, indicating incipient cell death processes (Figure 3B). With time and rising MTO concentrations, free or SPION-loaded, the proportion of apoptotic and later also necrotic cells increased. Here, no significant difference between free MTO and SPION^MTO^ was detected (Figure 3B, Figure S2a). Although after 24 h incubation the effects on cell viability were still weak, at this time the MTO-treated cells already exhibited alterations in the cell cycle as determined by PI-Triton staining (Figure 3C, Figure S2b). Because MTO intercalates into the DNA, inhibiting replication, cell cycle analysis showed earlier effects of MTO treatment. In MTO- and SPION^MTO^-treated cells the cell cycle phases shifted with increasing MTO dose from G1 to G2 phase after 24 h, reflecting cell cycle arrest in the G2 phase due to DNA damage. With prolonged incubation for 48 h, cells underwent apoptosis, accompanied by activation of DNases, which degraded the cellular DNA into smaller pieces (subG1 DNA) as seen in MTO- and SPION^MTO^-treated cells in a dose-dependent manner. After 72 h, cells treated with MTO in free or nanoparticle-loaded form are characterized by degraded DNA (Figure 3C, Figure S2b). So far, free and nanoparticle-loaded MTO have shown the same efficacy to induce cell death in HT-29 tumor cells.

### 3.4. Release of Damage-Associated Molecular Patterns (DAMPs) and Activation of Immune Cells

**Figure 4:**
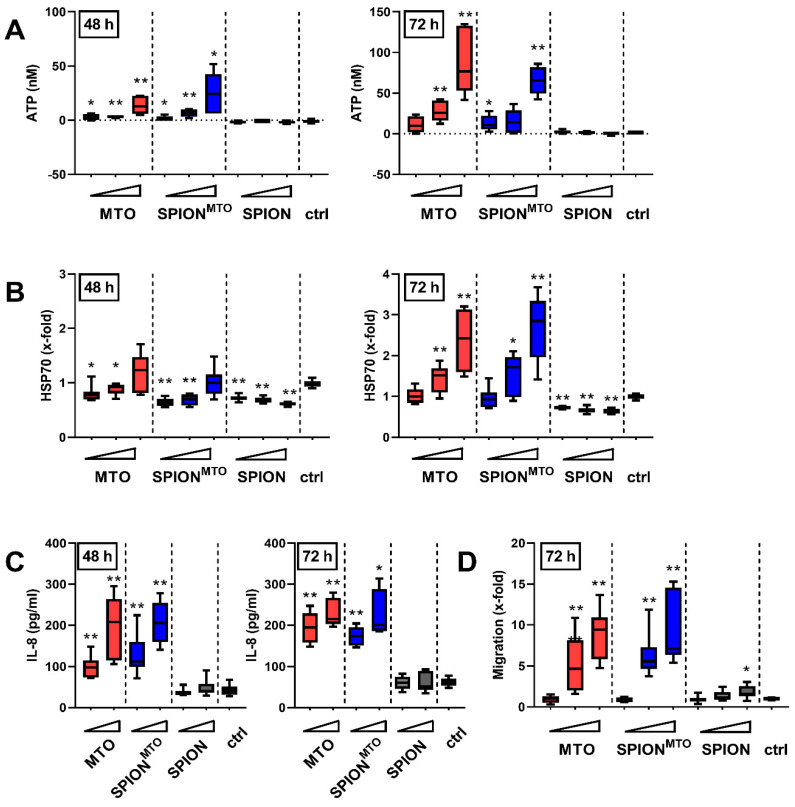
Release of damage-associated molecular patterns (DAMPs) from dying cells. The supernatants of cells which had been treated with SPION, MTO or SPION^MTO^ for 48 h or 72 h were analyzed via ATP-mediated chemiluminescence (**A**), HSP70 ELISA (**B**) or IL-8 bead-based immunoassay (**C**). (**A,B,D**) 0.5, 1.0 or 2.0 µM MTO and corresponding iron amounts were used according to Table 2 (see triangle); **(C)** 1.0 and 2.0 µM MTO were applied. (**D**) Supernatants were used as chemoattractant for THP-1 monocytes. Data represent number of THPs migrating towards the supernatant. The supernatant from H_2_O-treated cells served as control. Shown are the mean values and standard deviations of three independent (**A,B,D**) or two independent triplicates (**C**), respectively. Significances were calculated using Student’s *t*-test comparing treatment versus control group and MTO-treated cells versus SPION^MTO^-treated cells (* *p* < 0.05; ** *p* < 0.01).

Tumor cells killed with certain chemotherapeutics such as MTO can foster immune responses by releasing damage-associated molecular patterns (DAMPs). When analyzing supernatants of SPION-, MTO- or SPION^MTO^-treated cells, we detected ATP and HSP70 after 72 h incubation in a dose-dependent manner (Figure 4A,B). At this time, most of the cells already exhibited ruptured plasma membranes, indicating a post-mortem leakage induced both by free MTO or SPION^MTO^. At earlier measurement points, no (24 h, data not shown) or only a small amount of ATP or HSP70 was released (Figure 4A,B). In contrast, interleukin-8 (IL-8) was found in the supernatants as early as 48 h after treatment in high amounts which further increased until 72 h of incubation (Figure 4C). For SPION-treated cells, no release of ATP, HSP70 or IL-8 was detected. Altogether, MTO- and SPION^MTO^-treated cells showed a similar, dose- and time-dependent release of danger signals. To measure the capacity of the supernatants to attract immune cells, supernatants from HT-29 cells treated with MTO, SPION^MTO^ or SPION, respectively, were employed as chemoattractant for THP-1 monocytes in transwell assays and THPs migrating towards the supernatant were counted (Figure 4D). Supernatants from SPION-treated cells hardly induced migration, whereas supernatants from MTO-treated cells induced migration proportional to the administered amount of drug and released DAMPs. Again, there was no significant difference between MTO in its free or nanoparticle-bound form.

**Figure 5:**
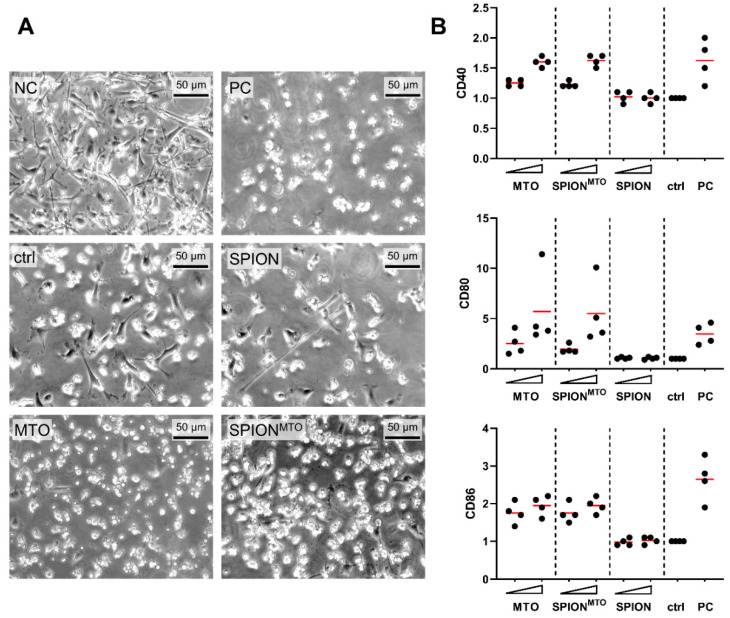
Maturation of dendritic cells after co-incubation with supernatants of HT-29 cells. Supernatants of HT-29 cells which had been treated for 72 h with SPION, MTO and SPION^MTO^ (MTO concentration 1.0 µM and 5.0 µM and corresponding SPION concentrations, see Table 2) were co-incubated with monocyte-derived immature dendritic cells (iDC) from four healthy donors. After 24 h of co-incubation, the expression of the activation markers CD40, CD80 and CD86 were analyzed using flow cytometry. Untreated iDC in HT-29 medium (NC), matured DC (mDC) in HT-29 medium (PC) and iDCs in supernatant from H_2_O treated HT-29 cells (ctrl) served as controls. (**A**) Light microscopic pictures of DCs incubated with supernatant of HT-29 cells treated with 1 µM MTO and corresponding concentrations of SPION and SPION^MTO^. (**B**) Each donor was tested at least as duplicate and each mean value was normalized to the untreated control. Every dot represents the mean value of one donor, the red line the mean of all donors.

For induction of an adaptive immune response, professional antigen presenting cells such as dendritic cells must ingest and process antigens and present them to effector cells. Thus, we wanted to analyze whether supernatants from SPION^MTO^-treated cells were able to induce maturation of dendritic cells. For this purpose, we co-incubated the supernatants with immature dendritic cells (iDC) and analyzed cell morphology and surface molecules. iDCs in the presence of SPION-treated supernatants showed the same behavior as the control group, with typical dendritic cell morphology and no induction of activation markers on the cell surface (Figure 5A,B). Supernatants from cells treated either with free or SPION-bound MTO on the other hand induced a spherical shape similar to the positive control group (PC) (Figure 5A) and an increase of cell activation markers CD40, CD80 and CD86 (Figure 5B) consistent with DC maturation (mDC) proportional to the initially applied MTO concentrations. Again, MTO loaded on SPIONs showed the same effect as the free drug.

## 4. Discussion

We showed that adherent HT-29 cells treated with SPION^MTO^ underwent cell cycle stop with concomitant degradation of the DNA. At the same time, apoptosis and later secondary necrosis were induced. Both MTO- and SPION^MTO^-caused cell death were accompanied by the release of ATP, HSP70 and IL-8 (Figure 4A–C) which acted as DAMPs, attracting and activating leukocytes (Figure 4D, Figure 5). Our experiments performed in HT-29 colon carcinoma cells confirmed previous data obtained from Jurkat T cells, showing that nanoparticle-loaded MTO provoked cell death with a comparable phenotype as its free counterpart, a prototypic ICD inducer [28]. In contrast to the free drug, MTO loaded onto SPIONs was magnetically accumulated in the desired area (Figure 2), while avoiding unwanted dispersion into peripheral regions. Importantly, the particles were not toxic towards erythrocytes nor did they induce unwanted plasma coagulation or platelet activation (Figure 1), a condition, amongst others, which constitutes a prerequisite for application in vivo into the blood stream. These data support earlier findings which show excellent biocompatibility of the employed SPION system [37,38]. Regarding blood coagulation, prothrombin time and thrombin time were not affected by SPION concentrations up to 0.5 mg/mL (Figure 1). In contrast, activated partial thromboplastin time was extended correspondingly to the amount of SPIONs employed (Figure 1), making further experiments necessary to investigate the effects of SPIONs on coagulation factors XII, XI, IX and VIII. So far, this observation is not regarded as an obstacle for application in magnetic drug targeting because previous in vivo experiments have shown that particles are magnetically attracted out of the vessels into the tumor region immediately and remain there even after the magnetic force has ceased [24]. On the contrary, the prevention of vascular occlusion represents an important point. Our SPIONs consisted of clusters (52–56 nm in hydrodynamic diameter) of individual nanoparticles, each smaller than 20 nm core size, being a prerequisite for superparamagnetism. Consequently, after switching off the magnetic field, the SPIONs do not form magnetic agglomerates, as has been verified by Zaloga et al. [38]. The stable and proper coating of the nanoparticles is mandatory for the prevention of agglomerate formations, as well. We previously showed that the magnetic agglomeration of SPIONs with inappropriate coating can foster the formation of neutrophil extracellular traps (NETs), which are prone to occluding vessels [39]. The coating with lauric acid and human serum albumin (as used in this study) has been shown to enable sufficient magnetic accumulation of the SPIONs but prevents the formation of NETs.

So far, conventional chemotherapy with systemically applied MTO requires high drug concentrations, which kill not only tumor cells, but also immune cells, causing immunosuppressive side effects and thereby inhibiting the ICD-induced immune response. To minimize adverse effects and to maximize the concentration of drug in the tumor region, magnetic drug targeting provides a promising solution [40]. Several attempts have already been made to enrich chemotherapeutic drugs within tumors with magnetic nanoparticulate transporter systems [41,42]. Since the targeted transport of MTO by SPIONs spares cells outside the stretch of the magnetic field (Figure 2), we suggest that cell death is only locally induced and immune cells, necessary for induction of immune reactions, are spared from chemotherapeutic burden when applied in vivo. We already showed accumulation of MTO and locally increased cell death induction in two- and three-dimensional cell culture under flow conditions using SPIONs as transporter system [28,43]. In the present study, we confirmed earlier findings of experiments performed in Jurkat cells [28] that not only free MTO but also its nanoparticle-loaded counterpart can induce the release of proinflammatory DAMPs (Figure 4A–C), which might eradicate the tumoral immune suppressive microenvironment. The chemotactic attraction of monocytes by supernatants of HT-29 cells, killed by SPION^MTO^ (Figure 4D), and activation of DCs (Figure 5) supports the suggestion that SPION^MTO^ induces ICD comparable to its free counterpart.

According to the consensus guideline for the identification of an ICD-inducing agent, three points should be addressed: first, the study of DAMP emission from the dying cells; second, tests to assess the activation of APCs in vitro; and third, the ability of dying cells to initiate adaptive immunity in vivo in immune-competent, syngeneic hosts [8,44]. In this study, after SPION^MTO^ treatment we verified the presence of DAMPs HSP70 and ATP in supernatants of dying HT29 cells and upregulation of activation markers CD80, CD83 and CD86 in DCs, which have been defined as in vitro surrogate markers for ICD. Previously, we had already detected the release of ATP, HMGB1, and HSP90 from dying Jurkat cells and exposition of calreticulin on the surface of early apoptotic ones induced by SPION^MTO^ [28].

We are aware that the gold standard, however, should be animal experiments because investigations of the development of adaptive immune responses need a complex in vivo immune environment. As requested by the consensus guidelines, two sets of experiments should be performed in vivo: first, vaccination assays, where cells are killed in vitro and injected subcutaneously into immune-competent mice to investigate their potential in inducing a protective anticancer immune response against a challenge with living tumor cells one week later. Second, therapeutic assays, where immune-competent and immune-deficient tumor-bearing mice are treated with the putative ICD inducer, followed by monitoring of the tumor size [8]. MTO itself is a bona fide inducer of ICD, which has been shown in a number of hallmark experiments [45,46,47]; however, loading the drug onto a transporter system did not change its mode of action of inducing cell death in all experiments we have performed so far [28,43,48,49]. Experiments in tumor-bearing mice exclusively showing that cells which have been killed by SPION^MTO^ in vitro can prevent tumor growth when challenged, however, have only limited additional value and contradict the 3 R (replace, reduce, refine) principle for animal experimentation. Immunogenically dying CT26 colon carcinoma cells killed by MTO in vitro and adorned with CpG nanoparticles (adjuvant TLR9-agonist) have been already shown to foster maturation of DCs, T cell responses and antitumor responses in mouse models [50]. With our approach using magnetic delivery of MTO by SPIONs, tumor cells might be in situ turned into antigen and danger signal releasing vaccines, skipping additional ex vivo manipulations. With docetaxel, also identified as an ICD-inducing agent, MDT has been performed previously in CT-26-tumor-bearing mice, enhancing therapeutic efficacy and survival compared to not targeted application [25].

Our in vivo therapeutic setting using an electromagnet with a maximum magnetic field of 1.01 T at the very tip of the pole show at a current of 72 A to magnetize the SPIONs was constructed for the use of larger organisms than mice, which makes the procedure more time-consuming, more expensive, enables only smaller group sizes and must be planned carefully. When we performed MDT with SPION^MTO^ in tumor-bearing rabbits previously, we observed that the tumor shrank over several weeks, which is more indicative of an immune-mediated effect than immediate tumor lysis [26]. We also showed that immune cells of the peripheral blood were spared from the toxic effects in this setting [27]. Advanced readouts and analyses with rabbit-derived cells will be a future challenge since most kits and antibodies available are designed for cells and fluids from mouse, rat and human. Established methods must be adapted accordingly to perform in vivo experiments verifying that after application of SPION^MTO^ and magnetic accumulation in tumor tissue, an immunogenic tumor response can be finally achieved by cytotoxic T cells.

Nanoparticles which can induce localized cell death with immunogenic properties might thus be used to turn immunologically classified “cold” tumors with low intratumoral lymphocyte infiltration rates into “hot” immune-infiltrated tumors [51,52] to enable subsequent immune interventions. Exemplarily, doxorubicin-loaded manganese dioxide nanoparticles have been accumulated by Amini et al. in the tumor via the EPR effect, thus abolishing the immunosuppressive environment in an orthotopic mouse model of breast cancer [53]. To overcome immune escape mechanisms of the tumor cells and increase treatment efficacy, nanoparticles such as SPIONs might be loaded with additional molecules such as checkpoint inhibitors, serving as a platform for multimodal tumor therapy [54].

## Figures and Tables

**Table 1:** Physicochemical properties of superparamagnetic iron oxide nanoparticles (SPION) and mitoxantrone-loaded ones (SPION^MTO^) adapted from [28,29], Elsevier, 2018, 2016.

Physicochemical Properties	Method	SPION	SPION^MTO^
Hydrodynamic size (nm) and polydispersity index	Dynamic light scattering (DLS) in RPMI 1640 (Malvern Zetasizer NanoZS)	52.7 ± 0.8 nm; 0.202 ± 0.026	55.7 ± 1.0 nm; 0.239 ± 0.019
Core size (nm)	Freeze fracture TEM	10 nm iron core size with 2 nm organic layer	n.d.
Zeta potential (mV)	Malvern Zetasizer NanoZS; in RPMI 1640 medium	−11.9 ± 0.8	−12.4 ± 0.5
Drug loading efficacy (%) (200 µg mitoxantrone (MTO) per 1 mL SPIONs)	High-performance liquid chromotograpy (HPLC)-UV of supernatant to detect unbound drug	98.9 ± 0.2%	
MTO release (%) after 72 h at 37 °C in RPMI 1640 medium	(1) Dialysis bag method (2) Magnetic assay	11.6 ± 0.1% 23.7 ± 0.4%	

**Table 2:** Amount of mitoxantrone (MTO) and iron in MTO, SPION^MTO^ and non-loaded SPIONs.

Substance	Free MTO				SPION^MTO^				SPION			
**MTO [µM]**	0.5	1	2	5	0.5	1	2	5	-	-	-	-
**MTO [µg/mL]**	0.22	0.44	0.88	2.2	0.22	0.44	0.88	2.2	-	-	-	-
**Fe [µg/mL]**	-	-	-		5.32	10.64	21.28	53.2	5.32	10.64	21.28	53.2

## Supplementary Materials

The following are available online at https://www.mdpi.com/1999-4923/12/10/923/s1, Figure S1: SPION and MTO uptake into the cells, Figure S2: Flow cytometry raw data files.
