# Supplementary material for: Mitoxantrone-Loaded Nanoparticles for Magnetically Controlled Tumor Therapy–Induction of Tumor Cell Death, Release of Danger Signals and Activation of Immune Cells
Source: Pharmaceutics. 2020 Sep 27;12(10):923. doi: 10.3390/pharmaceutics12100923 (PMC7599695; doi:10.3390/pharmaceutics12100923)
Supplement: Supplementary file 1 [file pharmaceutics-12-00923-s001.pdf]

pharmaceutics

Supplementary Materials: Mitoxantrone-Loaded Nanoparticles for Magnetically Controlled Tumor Therapy–Induction of Tumor Cell Death, Release of Danger Signals and Activation of Immune Cells

Teresa Ratschker, Laura Egenberger, Magdalena Alev, Lisa Zschiesche, Julia Band, Eveline Schreiber, Benjamin Frey, Anja Derer, Christoph Alexiou and Christina Janko

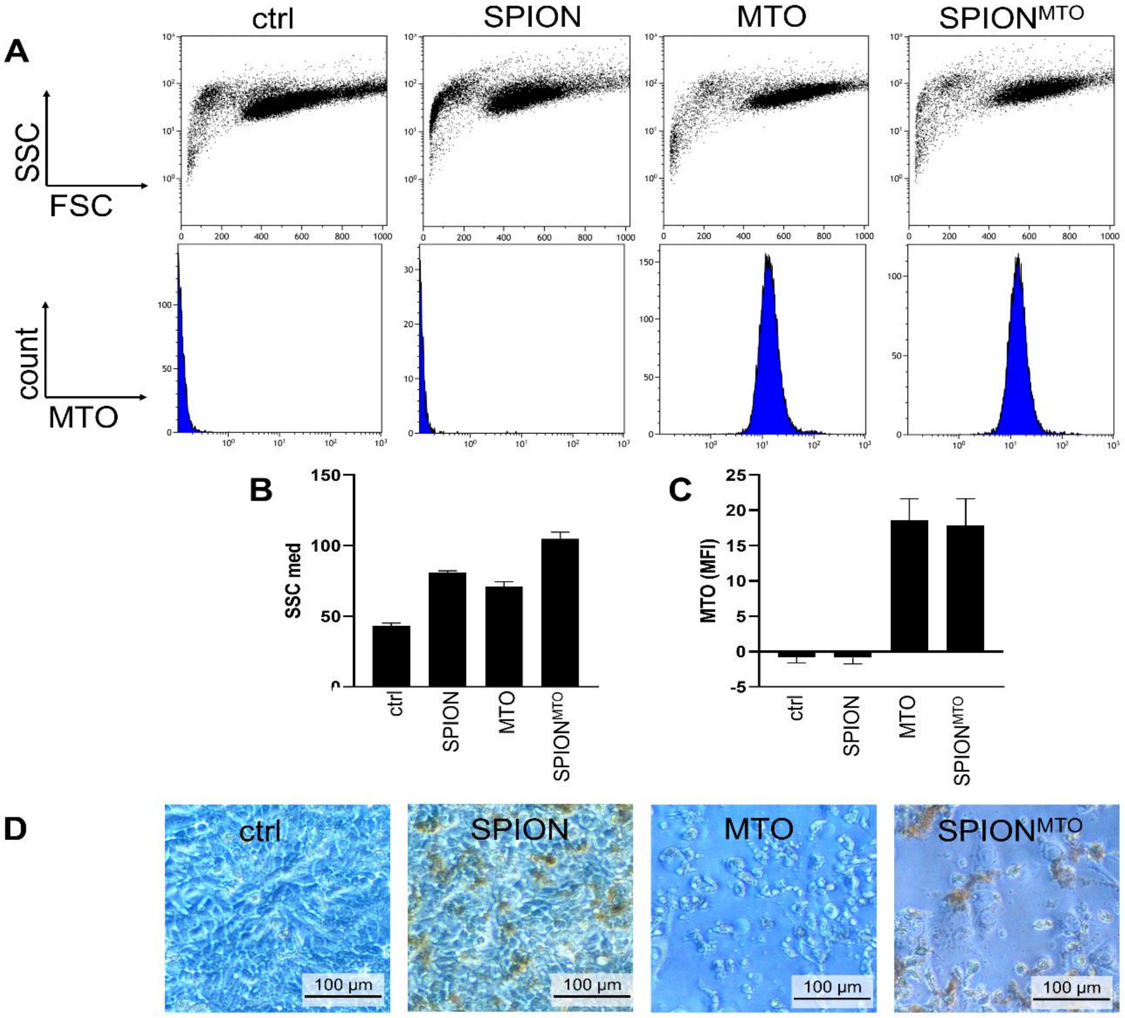

Figure S1. SPION and MTO uptake into the cells. (**A**) Flow cytometry raw data files of HT-29 cells after 24 h treatment with SPION, MTO or SPION^{MTO} (MTO concentration 2 µM and corresponding SPION concentration). H_2O-treated cells served as controls. Upper row: side scatter (SSC) against forward scatter (FSC), lower row: MFI of FL7 indicates intracellular MTO; (**B**) Side scatter (median values) of Ax-PI− cells; (**C**) intracellular MTO intensities (mean values) of Ax-PI− cells. (**B**,**C**) Analyzed cells were gated for viability (Ax-PI−). Shown are the median/mean values with standard deviations of one representative triplicate. (**D**) Brightfield microscopy of HT-29 cells after incubation for 72 h with SPION, MTO or SPION^{MTO}.

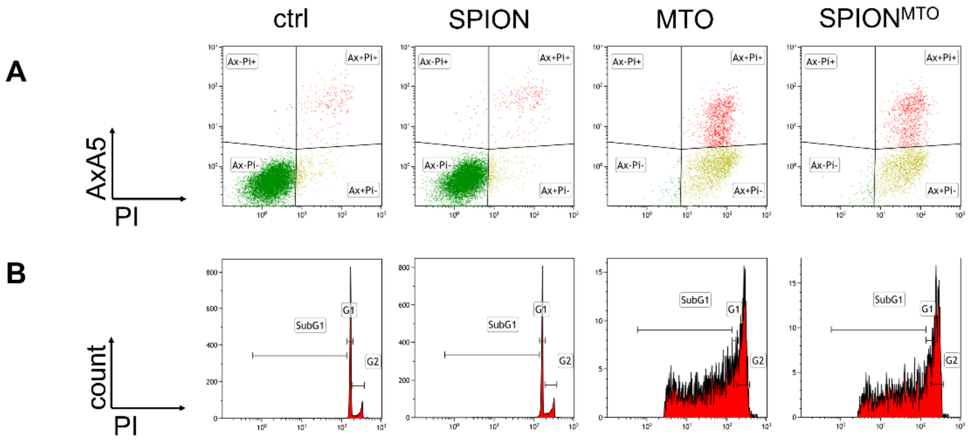

Figure S2. Flow cytometry raw data files. (**A**) AxPI staining and (**B**) PI-Triton staining for determination of cell viability and cell cycle, respectively. HT-29 cells were incubated for 72 h with SPION, MTO or SPION^{MTO} (MTO concentration 2 µM and equivalent SPION concentration). H_2O-treated cells served as control.
